# Efficacy of Moraceae with chlorhexidine mouthwash on the microbial flora of critically ill intubated patients: a randomized controlled pilot study

**DOI:** 10.1038/s41598-022-21556-y

**Published:** 2022-10-14

**Authors:** Pasu Siriyanyongwong, Rawee Teanpaisan, Nuntiya Pahumunto, Supattra Uppanisakorn, Veerapong Vattanavanit

**Affiliations:** 1 Division of Internal Medicine, Faculty of Medicine, Prince of Songkla University, Hat Yai, Songkhla, 90110 Thailand; 2 Department of Stomatology, Faculty of Dentistry, Prince of Songkla University, Hat Yai, Songkhla, 90110 Thailand; 3 Clinical Research Center, Faculty of Medicine, Prince of Songkla University, Hat Yai, Songkhla, 90110 Thailand; 4 Critical Care Medicine Unit, Division of Internal Medicine, Faculty of Medicine, Prince of Songkla University, 15 Kanjanavanich Road, Hat Yai, Songkhla, 90110 Thailand

**Keywords:** Microbiology, Diseases, Health care, Medical research

## Abstract

Critically ill intubated patients are routinely provided with chlorhexidine (CHX) for their mouth washing, but CHX mouthwash induces several complications. In this study, we aimed to evaluate the efficacy and safety of Moraceae with CHX mouthwash in the reduction of oral bacterial count in critically ill patients and to compare it with CHX-alone mouthwash. This double-blind, randomized, controlled trial included critically ill patients receiving mechanical ventilation. The patients were randomly divided into two groups based on the Modified Beck oral assessment score. The primary endpoint was a reduction in oral bacterial counts after mouth washing on day 1 and day 4. Thirty patients were included in this study; 15 patients received Moraceae with CHX mouthwash and 15 patients received CHX-alone mouthwash. The oral bacterial counts in the Moraceae with CHX group did not differ from the CHX group after mouth washing on day 1 and day 4 of admission. The patients in the CHX group experienced more intolerable taste than those in the Moraceae group (60% vs. 13.3%, *P* = 0.008). Moraceae with CHX mouthwash had the same effectiveness as CHX alone on bacterial flora but exhibited less intolerable side effects than CHX alone.

Trial registration: TCTR20190530003; 30/05/2019.

## Introduction

Ventilator-associated pneumonia (VAP) is one of the most critical problems in intensive care units (ICU) and is the most common nosocomial infection among patients receiving mechanical ventilation. The incidence of VAP is approximately 8–28%^1,2^. The mechanism includes bacterial oropharyngeal colonization that progresses into tracheal colonization that subsequently leads to pneumonia. A previous study reported that the causative pathogen was found in the oral cavity in the first 24 h^3^. When patients are intubated, the normal flora in the oral cavity is replaced by pathogens, thus causing VAP. The number of pathogens increases from the first day to the fourth day and continues to increase till 7 days^4,5^. In a Thai study, the major organisms were gram-negative bacteria such as *Acinetobacter baumannii, Klebsiella pneumoniae,* and *Pseudomonas aeruginosa* and the most common gram-positive pathogen was *Staphylococcus aureus*^6^. The mortality rate of patients with VAP is two times the mortality rate of patients without VAP^7^. The estimated attributable mortality of VAP is around 10% and the disease also increases hospitalization duration and cost^8^.

Antiseptic oral mouthwash such as chlorhexidine (CHX) was proposed as one intervention to reduce VAP. As CHX exhibits antiseptic effects against oral pathogens causing VAP, CHX mouthwash is becoming popular in ICU patients^9^. In a meta-analysis, CHX mouthwash decreased bacterial colonization and VAP^10^. However, the results of CHX are debatable and found to be dependent on the concentration of CHX^11,12^. At higher concentrations and extended periods of usage, CHX causes mucositis and oral ulcer, as well as changes in taste^13^.

An in-vitro study reported that *Artocarpus lakoocha* belonging to the family Moraceae exhibits oral antibacterial and antibiofilm effects^14^. This herb is commonly found in tropical regions such as Thailand and India. The oxyresveratrol as the major constituent of aqueous extraction of *Artocarpus lakoocha* is suggested to affect the integrity of bacterial cells^14^. A pilot study in healthy volunteers revealed that mouthwash products containing 0.02% Moraceae with 0.0005% CHX exhibit antimicrobial and antiplaque activities similar to 0.12% CHX mouthwash. Moreover, the CHX mouthwash group developed a burning sensation and distaste (unpublished data).

In this study, we aimed to evaluate the efficacy and safety of Moraceae with CHX mouthwash in reducing oral bacteria count in critically ill patients and to compare it with CHX alone. We hypothesized that Moraceae with CHX mouthwash has comparable antimicrobial activities with CHX mouthwash alone and is safe for use in critically ill intubated patients.

## Methods

### Study design

We performed a double-blinded, randomized clinical study of patients between May 2019 and October 2021 in the 10-bed medical ICU of Songklanagarind Hospital, Hat Yai, Thailand.

The study protocol was approved by the Institutional Review Board (REC 61-434-14-1) and registered in the Thai Clinical Trial Registry (TCTR20190530003; 30/05/2019). An independent data safety monitoring board monitored the study for complications with predetermined discontinuation criteria (presence of erythema of the mucosa or grade I of oral mucositis score according to the Radiation Therapy Oncology Group^15^ or conscious patients felt uncomfortable or patients denied). The study complies with all principles of the Declaration of Helsinki (1964) and its subsequent versions. Informed consent to participate was obtained from participants who met eligibility criteria before the initiation of the study or from their proxies.

### Patient selection

Patients aged 18 years or older who were admitted to the medical ICU within 24 h and expected to require mechanical ventilation for at least more than 72 h were included in the study. Exclusion criteria are shown as follows: (1). Intubation for more than 24 h, (2). Pregnancy or sensitivity of anaphylaxis to herbal preparation and CHX, and (3). Individuals with orthodontic appliances or prosthetic appliances that would interfere with evaluation.

### Randomization

The investigators evaluated patients for eligibility, obtained informed consent, and enrolled the eligible participants. After inclusion, the patients were randomly assigned without restriction in the block of 4 with a 1:1 ratio stratified by the Modified Beck oral assessment score (BOAS)^16^ (normal to mild (scores 5–10), moderate (scores 11–15), and severe dysfunction (scores 16–20); supplementary appendix) according to a computer-generated randomization table derived from www.randomization.com by a research nurse assistant, who had no role in patient management. The research nurse assistants who were not otherwise involved in the study administered the study mouthwash and CHX. The attending physicians, nursing care teams, research investigators, and participants and their family members were blinded to treatment allocation. The mouthwash was prepared by a pharmacist who had no other role in the trial. The mouthwashes were packaged in non-identical 10 mL bottles labeled with sequential numbers.

### Ethical approval and consent to participate

This study was performed according to the Helsinki Declaration and was approved by the Institutional Review Board (REC 61-434-14-1), the ethics committee of the Faculty of Medicine, Prince of Songkla University. Informed consent to participate was obtained from participants who met eligibility before study initiation or from their proxies.

## Study intervention

### Mouthwash preparation

For Moraceae mouthwash preparation, we used 0.02% *Artocarpus lakoocha* extract, 0.0005% CHX, and cremophor RH40 in the mouthwash base, which was prepared in the standard laboratory of the Faculty of Dentistry, Prince of Songkla University (Patent no. 9519). CHX mouthwash of 0.12% was prepared in the pharmaceutical unit of Songklanagarind hospital.

The two types of mouthwashes were straw-yellow colored and filled in non-identical, 10 mL bottles.

### Oral care

Before oral hygiene intervention, The Modified BOAS was recorded, which ranged between 5 and 20, with 5–10 representing normal to mild dysfunction, 11–15 representing moderate dysfunction, and 16–20 representing severe dysfunction. The first oropharyngeal suction (T0) in the lower gingivobuccal sulcus to quantify bacterial inoculum was performed after randomization. Subsequently, tooth brushing (Colgate) with toothpaste (Colgate) was performed by bedside nurses. The mouth washing of the first group was performed with 10 mL of Moraceae with CHX solution every 8 h. The mouth washing of the second group was performed with 0.12% CHX solution alone every 8 h. After mouth washing, the second sample (T1) was collected by oropharyngeal suction. Oral care with intervention mouthwashes was performed for 7 days or until the patients were extubated. The third sample (T2) was collected on the morning of day 4 before the oral care procedure.

The samples were collected immediately after mouth washing to avoid a decrease in the mouthwash salivary concentration and bacterial colonization^11^. Considering that the oral microflora changes to VAP pathogen at 48 to 72 h after hospitalization^5,17^, the samples were also collected on day 4. The Modified BOAS was recorded at 3–4 p.m. daily by the first author (PS). Patient satisfaction was surveyed at the end of the intervention in conscious patients.

### Microbiological study

The saliva samples were transferred to 1.5 mL sterile microcentrifuge tubes and stored on ice for a few hours for transportation to the research laboratories. The saliva samples were immediately stored at − 80 °C until used for real-time PCR. Saliva samples were collected at baseline (T0), after mouthwash (T1), and day 4 (T2), and evaluated for *Staphylococcus aureus, Pseudomonas aeruginosa, Acinetobacter baumannii, and Klebsiella pneumoniae* using real-time PCR.

### Microbial evaluation using real-time PCR

The whole salivary samples were collected from individual subjects at baseline, immediately after using mouthwash, and 4 days after using mouthwash by having them spit into sterile plasticware. DNA was extracted from the salivary samples using a PureDirex Genomic DNA Isolation kit (Bio-Helix Co., LTD., Keelung City, Taiwan) following the manufacturer’s protocol for salivary bacteria, and bacterial DNA was stored at − 20 ºC until further use. The quantities of targeted bacteria in the saliva at baseline (T0), immediately after using mouthwash (T1), and 4 days after using mouthwash (T2) were evaluated by performing real-time PCR. Total bacterial DNA (5 μL) was added to a Sensi-FAST™SYBR kit (Bioline Reagent Ltd., California, USA). The sequences of primers used were as follows: total bacteria (5′-TCCTACGGGAGGCAGCAGT-3′ and 5′-GGACTACCAGGGTATCTAATCCTGTT-3′)^18^, *A. baumannii* (5′-CATTATCACGGTAATTAGTG-3′ and 5′-AGAGCACTGTGCACTTAAG-3′)^19^, *K. pneumoniae* (5′-AGAGTATTGGTTGACTGCAGGATTT-3′ and 5′- AAACATCAAGCCATATCCATTGG-3′)^20^, *P. aeruginosa* (5′- CCTGACCATCCGTCGCCACAAC-3′ and 5′-CGCAGCAGGATGCCGACGCC-3′)^21^, and *S. aureus* (5′-GAAATCGATGGTGACAGTAATAA-3′ and 5′- CTACGTCATTTGCACCYGATAA-3′)^22^. The PCR cycles consisted of an initial step of 10 min at 95 °C followed by 2 min at 50 °C and 40 cycles at 95 °C for 20 s with different annealing temperatures, including 60 °C for total bacteria and *P. aeruginosa*, 59 °C for *K. pneumoniae*, 57 °C for *S. aureus*, and 52 °C for *A. baumannii* for 20 s. The polymerization temperature was 72 °C for 25 s. Amplification, detection, and data analysis were performed using the CFX96™ Real time system (Bio-Rad Laboratories, Inc., Hercules, CA, USA). Each sample was run in duplicate and the number of targeted bacteria in the saliva samples was quantified using a standard curve.

The standard curve of each target bacteria has been previously reported^23^. The bacterial pellets of the target bacteria were collected after centrifugation at 3000* g* for 5 min, washed twice with phosphate-buffered saline (pH 7.0), and adjusted to OD_600nm_ = 2.0 using a UV/VIS spectrophotometer (Biochrom Ltd., Cambridge, UK). The bacterial cell suspensions were two-fold diluted, which were then divided into two aliquots. To determine the bacterial number as colony-forming units (CFU)/mL, the first aliquot was measured using the cultivation method. The second aliquot was used for DNA extraction, as mentioned above, to determine the quantification cycle (Cq) of real-time PCR using the CFX96 Touch™ Real-Time PCR detection system (Bio-Rad Laboratories). A linear standard curve was plotted for each bacterial species from log CFU/mL against the corresponding Cq, showing a high correlation coefficient (R^2^ > 0.99).

### Study endpoints and data collection

The primary endpoint of the study was a reduction in oral bacterial total counts as indicated by CFU after mouthwash exposure. We used the following common pathogens: *A. baumannii*, *K. pneumoniae*, *P. aeruginosa*, and *S. aureus*.

The secondary endpoints included oral health using the Modified BOAS, patients’ satisfaction, and VAP during admission. Patients’ satisfaction evaluation was performed according to the clinical assessment reported by Sarvizadeh et al.^24^. The conscious patients were asked whether they experienced pain or discomfort during and after mouth washing and graded as tolerable and intolerable. VAP diagnosis was based on the Centers for Disease Control and Prevention criteria^25^.

Baseline data included age, gender, comorbidities, the reason for ICU admission, SOFA score, antibiotic use, mechanical ventilator days, and length of ICU stay. For adverse events related to intervention mouthwash, we monitored the signs of mucositis, gingivitis, or anaphylaxis.

All patients were followed up until discharge or death, whichever occurred first.

### Statistical analysis

The primary outcome was a reduction in total CFU over time after mouth washing in each group. An a priori effect size was difficult to determine because of the lack of appropriately precise previous data on which the calculation was based. According to the report by Julious SA^26^, the appropriate sample size for a pilot study is 12 participants per group. With a 20% dropout rate consideration, we planned to enroll a total of 30 patients. There was no planned interim analysis.

The study was analyzed on an intention-to-treat basis. No imputation was performed. The Shapiro–Wilk test was used to assess the normal distribution of continuous variables. Continuous data are expressed as the mean and standard deviation or median and interquartile range, depending on data distribution. Numbers and percentages were used to describe the categorical variables. The differences in patient characteristics and outcomes between the two groups were compared using the Wilcoxon-rank sum test, Fisher’s exact test, and χ^2^ test, as appropriate.

Repeated-measures outcomes such as bacterial counts from patients’ saliva and Modified BOAS at different times were compared between the groups by the GEE with the population-average model. The working correlation matrix structure for GEE analysis was guided by the lowest quasi-likelihood under the independence model criterion (QIC). Finally, the interaction of the group with time was reported as the β-coefficient and 95% Wald confidence interval (CI).

A *P* value of < 0.05 was considered statistically significant for all comparisons. All statistical analyses were performed using STATA version 16 (StataCorp, College Station, TX, USA) and SigmaPlot (Systat Software, San Jose, CA).

## Results

### Patients’ flow and recruitment

**Figure 1:**
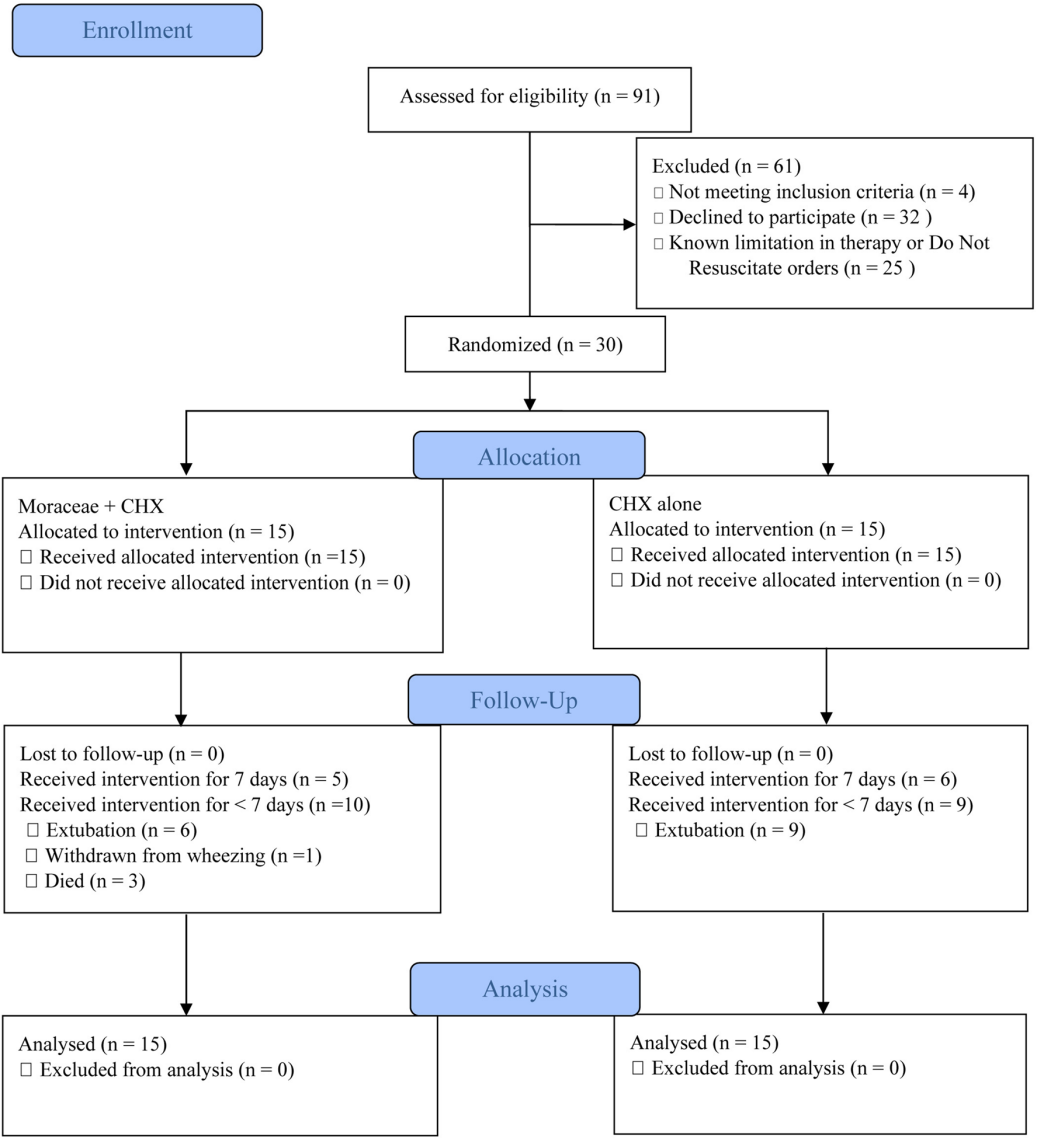
Flow diagram describing the screening, recruitment, and randomization of patients. CHX, chlorhexidine.

A total of 30 patients were recruited randomly for this study (Fig. 1). Fifteen patients were assigned to the Moraceae group and 15 patients were assigned to the CHX group.

### Baseline characteristics

The baseline characteristics of patients were comparable between the two groups Table 1. In all groups, the majority of patients were male; the median age ranged from 56 to 75 years. The two groups did not differ significantly in terms of comorbidities, sequential organ failure assessment (SOFA) score on admission, or reason for ICU admission. The majority of patients (73.3%) had initial normal to mild oral health status. Most patients received at least one antibiotic. Both the median mechanical ventilator and length of ICU stay were 5.5 days.

**Table 1:** Baseline characteristics of the patients.

Variables	Total (n = 30)	Group		*P*-value
		Moraceae + CHX (n = 15)	CHX (n = 15)	
**Demographic data**				
Sex (male)	16 (53.3)	8 (53.3)	8 (53.3)	1.000
Age (years)	65.5 (56–75.2)	68 (56–76)	63 (56–75)	0.561
**Underlying diseases**				
Malignancy	3 (10)	1 (6.7)	2 (13.3)	0.543
Cirrhosis	2 (6.7)	1 (6.7)	1 (6.7)	1.000
Chronic kidney disease	11 (36.7)	5 (33.3)	6 (40.0)	0.705
COPD	4 (16.1)	1 (6.7)	3 (20)	0.598
HIV	1 (3.3)	0	1 (6.7)	1.000
Chronic heart failure	7 (23.3)	5 (33.3)	2 (13.3)	0.390
**Reasons for ICU admission**				
Acute hypoxemic respiratory failure	12 (40)	7 (46.7)	5 (33.3)	0.456
Post cardiac arrest	10 (33.3)	7 (46.7)	3 (20.0)	0.121
Septic shock	20 (66.7)	8 (53.3)	12 (80.0)	0.121
Cardiogenic shock	4 (13.3)	3 (20)	1 (6.7)	0.598
SOFA	8.5 (4.8–11)	9 (7–11)	8 (4–10)	0.478
**Initial oral health score**^**a**^				
Normal to mild	22 (73.3)	11 (73.3)	11 (73.3)	0.598
Moderate dysfunction	4 (13.3)	1 (6.7)	3 (20.0)	0.598
**Ongoing exposure to antibiotic therapy**	29 (96.7)	14 (93.3)	15 (100)	1.000
3rd generation cephalosporin	15 (50.0)	8 (53.3)	7 (46.7)	0.715
Piperacillin-tazobactam	10 (33.3)	6 (40)	4 (26.7)	0.439
Carbapenem	15 (50.0)	7 (46.7)	8 (53.3)	0.715
Vancomycin	5 (16.7)	1 (6.7)	4 (26.7)	0.330
Fluoroquinolones	12 (40.0)	4 (26.7)	8 (53.3)	0.136
Ventilator days	5.5 (3–12.2)	5 (3–12)	8 (5–13)	0.112
ICU-length of stay (days)	5.5 (4–10.5)	5 (4–9)	6 (4–14)	0.287
General ward length of stay (days)	9 (3.5–20.5)	6 (2–14)	11 (5–22)	0.394
In-hospital mortality	9 (29)	6 (40)	3 (20.0)	0.193

Data are presented as the median (interquartile range) or n (%).

CHX, chlorhexidine; COPD, chronic obstructive pulmonary disease; HIV, human immunodeficiency virus; ICU, intensive care unit; SOFA, sequential organ failure assessment.

^a^Missing data of 4 patients were due to technical errors and lost files.

## Outcomes

### Oral microflora

Based on the real-time polymerase chain reaction (PCR) analysis, the value of bacterial counts was reported in log CFU/mL. Average total bacterial count and specific pathogens (*A. baumannii*, *K. pneumoniae*, *P. aeruginosa*, and *S. aureus*) at different times with the pairwise comparison between the groups are shown in Table 2.

**Table 2:** Microbial count comparison of mouthwash groups at the baseline, immediately after, and on day 4.

Organisms	Moraceae + CHX		CHX		*P*-value
	N	Log CFU/mL	N	Log CFU/mL	
**Total bacteria**					0.317
Before mouthwash (T0)	14	9.68 (1.11)	15	9.12 (1.21)	0.190
After mouthwash (T1)	14	8.51 (1.26)	15	8.79 (1.08)	0.359
Day 4 (T2)	7	9.77 (1.06)	8	9.01 (1.19)	0.297
***A. baumannii***					0.219
Before mouthwash (T0)	14	4.22 (1.28)	15	4.64 (1.69)	0.631
After mouthwash (T1)	14	2.49 (1.87)	15	2.77 (1.30)	0.555
Day 4 (T2)	7	3.16 (0.88)	8	3.19 (0.87)	0.954
**P. aeruginosa**					0.804
Before mouthwash (T0)	14	6.80 (2.15)	15	6.77 (1.33)	0.711
After mouthwash (T1)	14	4.39 (2.31)	15	4.41 (1.69)	0.965
Day 4 (T2)	7	5.40 (2.89)	8	5.14 (0.88)	0.908
***S. aureus***					0.341
Before mouthwash (T0)	14	5.35 (1.49)	15	5.78 (1.85)	0.541
After mouthwash (T1)	14	3.66 (1.53)	15	4.17 (1.47)	0.445
Day 4 (T2)	7	4.80 (1.28)	8	4.59 (1.45)	0.487
**K. pneumoniae**					0.879
Before mouthwash (T0)	14	6.97 (1.13)	15	7.07 (0.74)	0.776
After mouthwash (T1)	14	5.41 (0.89)	15	5.42 (1.28)	0.585
Day 4 (T2)	7	6.01 (1.19)	8	6.07 (0.79)	0.817

Data are presented as the mean (standard deviation).

^a^Missing value in T0 because of inadequate sampling for the microbial study.

^b^P-value; over time in each subgroup using generalized estimating equation and in pairwise time using Student t-test.

For the repeated-measures analysis by generalized estimating equations (GEE), the independent correlation matrix was eventually selected into the equation indicated by the lowest QIC. The results showed that total bacterial count and specific pathogen counts between the two groups were comparable (*P* > 0.05).

**Figure 2:**
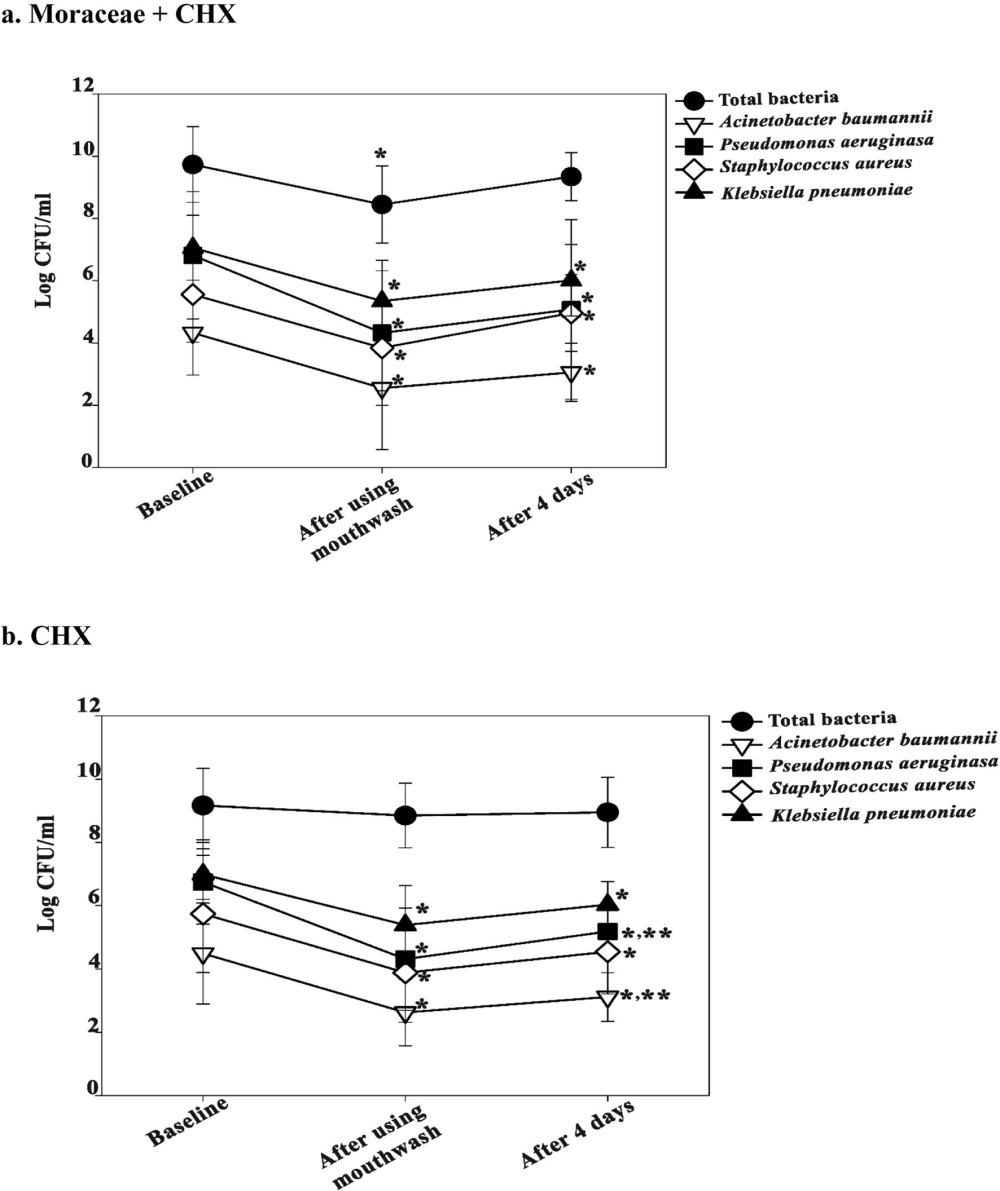
Bacterial counts in the mouthwash-treated patients’ saliva at the baseline, immediately after, and on day 4 **a**. Moraceae with CHX group **b**. CHX group. ^*^Statistically significant difference between T0 and T1 or T2 (*P* < 0.01). ^**^Statistically significant difference between T1 and T2 (*P* < 0.01). CHX, chlorhexidine; T0, baseline; T1, after using mouthwash; T2, after 4 days.

The bacterial count significantly decreased from baseline (T0) to immediately after mouthwash (T1) and increased on day 4 (T2) but was lower than baseline. In the CHX group, *P. aeruginosa* and *A. baumannii* counts in T2 significantly increased from those in T1 Fig. 2.

### Oral health

No significant difference was observed in the oral health status according to Modified BOAS for 7 days (β-coefficient of 0.41 (95% Wald CI: − 0.06 to 0.88)) Table 3. However, the patients in the Moraceae group had better oral health than that in the CHX group on days 5–7.

**Table 3:** Comparison of modified BOAS from days 1–7 between the two groups.

Day	Moraceae + CHX		CHX		*P*-value^a^
	N	Scores	N	Scores	
1^b^	12	9 (8–9.8)	14	9 (7.8–10.2)	0.895
2	9	9 (8–10)	14	9.5 (7.8–10.2)	0.604
3	8	10 (7.5–10)	12	9 (8.2–10)	0.968
4	7	10 (7–10)	7	9 (9–10)	0.788
5	6	9.5 (7.8–10)	7	10 (8–11)	0.326
6	5	9 (7.5–10)	7	10 (8–11)	0.271
7	5	9 (7.5–9.5)	6	10 (8–12)	0.191
Total					0.084*

Data are presented as the median (interquartile range).

BOAS, Beck oral assessment score; CHX, chlorhexidine.

^a^P-value; using generalized estimating equation with the independent correlation matrix.

^b^Missing modified BOAS data on day 1 because of a technical error in data collection.

### Patients’ satisfaction, VAP, and adverse events

Patients in the CHX group reported more intolerable taste (9/15, 60%) than those in the Moraceae group (2/15, 13.3%) with a significant difference (*P* = 0.008). The incidence of VAP was 20% overall. Table 4.

**Table 4:** Patients’ satisfaction and VAP data between 2 groups.

Outcomes	Total (N = 30)	Moraceae + CHX (n = 15)	CHX (n = 15)	*P*-value
**Patients’ satisfaction**				
Tolerable	5 (16.6)	4 (26.7)	1 (6.7)	0.330
Intolerable	11 (36.7)	2 (13.3)	9 (60)	0.008
Undetermined	14 (46.7)	9 (60)	5 (31.3)	0.143
VAP incidence	6 (20.0)	3 (20)	3 (20)	1.000
**VAP pathogens**				
*S. aureus*	0	0	0	NA
*K. pneumoniae*	2 (6.7)	1 (6.7)	1 (6.7)	1.000
*A. baumannii*	1 (3.3)	0	1 (6.7)	1.000
*P. aeruginosa*	1 (3.3)	1 (6.7)	0	1.000
Others^a^	4 (13.3)	1 (6.7)	3 (20)	0.400

Data are presented as n (%).

CHX, chlorhexidine; VAP, ventilator-associated pneumonia.

^a^Other organisms: *Stenotrophomonas multophila*, n = 3; *Candida albicans,* n = 1.

A patient in the Moraceae group had a hyperresponsive airway without definite cause during mouth washing and weaning from the ventilator and was withdrawn from the study for safety reasons. No major adverse events were reported related to the intervention.

## Discussion

This clinical study showed that mouth washing using Moraceae with CHX mouthwash reduced the bacterial load in the saliva, similar to CHX alone. The patients in the CHX group experienced more intolerable pain than those in the Moraceae group.

The mechanism by which Moraceae with CHX mouthwash reduced oral microbial count needs to be elucidated. Oxyresveratrol, the major constituent of *A. lakoocha* extract, might affect the cell wall integrity of bacterial cells. In a previous study, Moraceae revealed good antibacterial activity against both cariogenic bacteria and periodontopathogens with more susceptibility toward gram-negative bacteria than gram-positive bacteria^14^. These properties support our results that the total bacterial count decreased significantly after using mouthwash in the Moraceae with CHX group, which was, however, not observed in the CHX group. Moreover, CHX alone showed an increased count of *P. aeruginosa* and *A. baumannii* from day 1 to 4, which was not seen in the Moraceae with CHX group.

Oral chlorhexidine is widely used in critically ill patients to prevent the incidence of VAP based on the previous meta-analysis^27^. However, two subsequent meta-analyses revealed that CHX might cause excess mortality in ICU patients while failing to prevent VAP^12,28^. Moreover, a meta-analysis of 11 randomized controlled trials revealed that herbal mouthwashes have potential benefits in controlling plaque and inflammation, which was similar to the effects of CHX^29^. The most frequently used herb- or plant-derived constituent reported in the studies was green tea extract, followed by neem and marigold. Nevertheless, all studies focused on healthy participants having gingivitis, and no study intervention used mouthwash containing constituents from *A. lakoocha*. A systematic review of a herbal oral care product in critically ill patients involved 18 studies with more than 10 major natural products that showed the effects of reduction of the oral microbial flora comparable to CHX^30^.

Complications associated with CHX included the change of taste, dryness, and burning of the mouth leading to an intolerable feeling. The oral health status of patients in the Moraceae group showed a better trend than that in the CHX-alone group based on the Modified BOAS score. In the Moraceae group, we diluted CHX approximately 240 times, which could reduce unacceptable complications caused by the high concentrations of CHX. Two possible reasons behind no significant results are: (1). The baseline oral health status of the majority of patients was normal or mild dysfunction. (2). The median time of mouthwash usage was 5 days. On the other hand, the median time from using CHX to the onset of oral lesions was 8 days in another study^13^.

Our intervention did not affect VAP prevention. However, the incidence of VAP was nearly 20% in both groups. The possible explanation for the slightly high VAP incidence despite daily mouthwash and systemic antibiotics usage might be from non-strictly VAP prevention bundles, especially handwashing. The majority of pathogens of VAP were gram-negative bacteria. These results are in line with a study performed in Northern Thailand, which reported that the most common VAP pathogens are gram-negative bacteria^6^. The increase in oral flora on day 4 indicated that the critical care team needed to pay more attention to VAP prevention.

To the best of our knowledge, this is the first clinical study of Moraceae mouthwash intervention in critically ill patients. We collected data on both microbiological and clinical outcomes. Although this study was a pilot study, our randomized, concealed, double-blinded trial had rigid inclusion and exclusion criteria, high protocol adherence, and no loss to follow-up.

This study has some limitations. First, the study is single-centered and includes a small sample size. The latter is the main drawback of our study since only 7 and 8 patients remained for the microbial study on day 4 in the two groups, respectively. Further clinical investigations with a larger study population would provide a better positive correlation among Moraceae mouth washing benefits, bacterial flora reduction, and VAP incidence. Second, we studied only VAP pathogens; hence, we had no data on other types of bacteria. Third, we did not collect data on the sufficient dose of antibiotic used. If antibiotics of sufficient doses for gram-negative bacteria are applied in the treatment, the efficacy of mouthwash is possibly hidden or weakened. However, the types of antibiotics used did not differ between the two groups.

## Conclusions

Moraceae with CHX mouthwash had the same effectiveness as CHX alone on bacterial flora but exhibited less intolerable side effects than CHX alone in critically ill patients with mechanical ventilation.

## Supplementary Information

Supplementary Information.

## Data Availability

The data from this study are available from the corresponding author upon request.

## Abbreviations

VAP: Ventilator-associated pneumonia
ICUs: Intensive care units
CHX: Chlorhexidine
PCR: Polymerase chain reaction
CFUs: Colony-forming units
Cq: Quantification cycle
BOAS: Beck oral assessment score
SOFA: Sequential organ failure assessment
GEE: Generalized estimating equations
QIC: Quasi-likelihood under the independence model criterion
CI: Confidence interval
