# Supplementary material for: Efficacy of Moraceae with chlorhexidine mouthwash on the microbial flora of critically ill intubated patients: a randomized controlled pilot study
Source: Sci Rep. 2022 Oct 14;12:17261. doi: 10.1038/s41598-022-21556-y (PMC9568567; doi:10.1038/s41598-022-21556-y)
Supplement: Supplementary file 1 — Supplementary Information. [file 41598_2022_21556_MOESM1_ESM.docx]

**Title:** Efficacy of Moraceae with chlorhexidine mouthwash on the microbial flora of critically ill intubated patients: a randomized controlled pilot study

**Authors:** Pasu Siriyanyongwong^1^, Rawee Teanpaisan^2^, Nuntiya Pahumunto^2^, Supattra Uppanisakorn^3^, Veerapong Vattanavanit^4,*^

**Author affiliations**

^1^Division of Internal Medicine, Faculty of Medicine, Prince of Songkla University, Hat Yai, Songkhla, 90110, Thailand

^2^Department of Stomatology, Faculty of Dentistry, Prince of Songkla University, Hat Yai, Songkhla, 90110, Thailand

^3^Clinical Research Center, Faculty of Medicine, Prince of Songkla University, Hat Yai, Songkhla, 90110, Thailand

^4^Critical Care Medicine Unit, Division of Internal Medicine, Faculty of Medicine, Prince of Songkla University, Hat Yai, Songkhla, 90110, Thailand

**^*^Correspondence:** Veerapong Vattanavanit, MD

Critical Care Medicine Unit, Division of Internal Medicine, Faculty of Medicine, Prince of Songkla University, 15 Kanjanavanich Road, Hat Yai, Songkhla 90110, Thailand

Telephone number: +66848456228 Fax number: +6674429385

Email: [vveerapong@gmail.com](mailto:vveerapo@medicine.psu.ac.th)

**Table S1. Modified Beck Oral Assessment Scale**

| **Area** | **Score** | | | |
| --- | --- | --- | --- | --- |
|  | **1** | **2** | **3** | **4** |
| Lips | Smooth, pink, moist, and intact | Slightly dry, red | Dry, swollen isolated blisters | Edematous, inflamed blisters |
| Gingiva and oral mucosa | Smooth, pink, moist, and intact | Pale, dry, isolated lesions | Swollen red | Very dry and edematous, inflamed |
| Tongue | Smooth, pink, moist, and intact | Dry, prominent papillae | Dry, swollen, tip and papillae are red with lesions | Very dry, edematous, engorged coating |
| Teeth | Clean no debris | Minimal debris | Moderate debris | Covered with debris |
| Saliva | Thin, watery plentiful | Increase in amount | Scanty and somewhat thicker | Thick and ropy, viscid or mucid |
| Total Score | 5 No dysfunction | 6–10 Mild dysfunction | 11–15 Moderate dysfunction | 16–20 Severe dysfunction |
